# Health-Related Quality of Life in Menopausal Women with Cancer: Results from the CALCAN Study

**DOI:** 10.3390/cancers18061019

**Published:** 2026-03-21

**Authors:** Ana Cristina Ruiz Peña, Laura Baquedano Mainar, Pluvio J. Coronado Martín

**Affiliations:** 1 Department of Gynecology, Miguel Servet Hospital, 50009 Zaragoza, Spain; 2 School of Medicine, University of Zaragoza, 50009 Zaragoza, Spain; 3 Department of Gynecology, Hospital San Carlos, 94070 Madrid, Spain; 4 School of Medicine, Complutense University of Madrid, 28040 Madrid, Spain

**Keywords:** menopause, health-related quality of life, cancer survivorship, sexual health, depression

## Abstract

Background: Menopausal symptoms can negatively affect health-related quality of life (HRQoL), especially in women with a history of cancer. This study compared menopause-specific HRQoL in peri- and postmenopausal women with and without cancer and explored differences by cancer type, menopause treatment use, and depression. Methods: We performed a cross-sectional multicenter study using self-reported data from 6833 women enrolled through the *Mi Menopausia* mobile app between 2021 and 2024. HRQoL was assessed with the Cervantes SF-16 scale. Results: The final sample consisted of 6833 women: no cancer (*n* = 6482) and cancer (*n* = 351), further classified as gynecologic (*n* = 210) and non-gynecologic (*n* = 141). Cancer history was associated with worse HRQoL in the Sexuality domain (51.2 ± 23.8 vs. 48.3 ± 24.6; *p* = 0.013), while global HRQoL did not differ significantly between women with and without cancer (30.6 ± 21.7 vs. 32.3 ± 20.7; *p* = 0.130). Among cancer women, Sexuality scores were worse in non-gynecologic versus gynecologic cancers (55.7 ± 22.9 vs. 48.2 ± 24.1; *p* = 0.005). Depression was consistently associated with worse HRQoL in all groups, while menopause treatment use was associated with poorer HRQoL only in women without cancer. Conclusions: Cancer history was mainly associated with poorer sexual menopause-related HRQoL rather than global HRQoL. Depression was a major factor linked to impaired HRQoL, highlighting the need for integrated sexual and mental health assessment in menopausal women, particularly cancer survivors.

## Simple Summary

Menopause can impair quality of life by affecting physical, emotional, and sexual well-being, with potentially greater impact in women with a history of cancer. Using data from a large app-based cohort, we compared menopause-related quality of life in peri- and postmenopausal women with and without cancer, including differences by cancer type. Cancer history was mainly linked to poorer sexual quality of life, while depression was consistently associated with worse outcomes across all groups, supporting more integrated menopause care with greater attention to sexual and mental health.

## 1. Introduction

Menopause and the menopausal transition are frequently accompanied by vasomotor symptoms, sleep disturbance, musculoskeletal pain, mood changes, and sexual complaints, which collectively impact women’s health-related quality of life (HRQoL) [1,2,3,4]. In cancer survivors, menopausal symptom burden and HRQoL impairment may be further exacerbated by treatment-induced ovarian insufficiency, abrupt hypoestrogenism, surgical menopause, endocrine therapies, and persistent physical and psychological sequelae [5,6]. Sexual health is particularly vulnerable due to genitourinary syndrome of menopause, dyspareunia, body image changes, and relationship-related factors; accordingly, sexual dysfunction is increasingly recognized as a core survivorship outcome, including in gynecologic oncology [7,8].

As population ageing and advances in early detection and treatment increase cancer survivorship, the clinical focus is progressively shifting from survival alone to long-term wellbeing and patient-reported outcomes, highlighting the growing recognition that, among cancer survivors, quality of life may be more important than the quantity of survival time. However, evidence regarding menopause-specific HRQoL across different cancer types remains heterogeneous, and large community-based real-world datasets are limited [6,9,10]. Digital health platforms can complement traditional clinic-based research by enabling large-scale assessment of symptoms and HRQoL in routine life settings, potentially reducing barriers to participation and capturing patient experiences outside specialized services [11,12].

The Cervantes SF-16 scale is a validated menopause-specific HRQoL instrument widely used in Spanish populations. It evaluates key menopause-related domains including Menopause-Health, Psychological, Sexuality, and Partner, and provides a global score that supports both clinical interpretation and research comparability [13,14,15].

Despite growing recognition of survivorship-related menopausal burden, important knowledge gaps remain. In particular, menopause-specific HRQoL has been less frequently examined than general survivorship outcomes, and direct comparisons between women with and without a history of cancer are still scarce, especially in large real-world community samples [10,16,17]. Moreover, differences according to cancer type and the contribution of potentially modifiable factors such as depression and menopause treatment use remain insufficiently characterized. Addressing these gaps is particularly relevant in the current context of increasing cancer survivorship and population ageing, as it may help inform more personalized and integrated approaches to menopause care [4,16].

The principal aim of this study is to compare menopause-specific HRQoL (Cervantes SF-16) between peri- and postmenopausal women with and without a history of cancer using data from a large app-based cohort. We further explored HRQoL differences according to cancer type (gynecologic vs. non-gynecologic) and examined the associations of menopause treatment use and depression with HRQoL.

## 2. Materials and Methods

### 2.1. Study Design

This was a multicenter, real-world, app-based study conducted in Spain using self-reported data collected through the Mi Menopausia mobile application, developed by the Spanish Association for the Study of Menopause (AEEM) to assess menopause-related symptoms and health-related quality of life (HRQoL) in real-world settings.

### 2.2. Study Setting and Data Collection

The study population comprised peri- and postmenopausal women who used the Mi Menopausia app between June 2021 and June 2024. Participants were recruited through complementary channels. First, gynecologists and other healthcare professionals recommended the *Mi Menopausia* app during clinical consultations as a tool for systematic symptom tracking and quality-of-life monitoring. Second, the app was promoted through social media and AEEM educational activities, after which women downloaded the app and self-completed the questionnaires on their own devices.

Menopausal status was defined using STRAW-based operational criteria [18]. Postmenopause was defined as ≥12 months since the last menstrual period or bilateral oophorectomy. In women with hysterectomy without bilateral oophorectomy, postmenopause was assigned based on compatible symptomatology and clinical context (e.g., oncologic treatment, age > 55 years, or clinical confirmation). Perimenopause was defined as <12 months since the last menstrual period, irregular cycles with amenorrhea ≥ 60 days, and climacteric symptoms.

Eligible participants were peri- or postmenopausal women according to the study’s operational definitions, able to read Spanish and provide informed consent, capable of using a mobile application, and who fully completed all questionnaires. A total of 7103 app records were screened; 270 were excluded due to incorrect, incomplete, unclear, or duplicate entries, resulting in 6833 eligible peri- and postmenopausal women.

Cancer history was self-reported and participants were classified as having no cancer (*n* = 6482) or cancer (*n* = 351). The cancer group was further categorized into gynecologic cancer (*n* = 210) and non-gynecologic cancer (*n* = 141). Baseline questionnaires captured sociodemographic variables, lifestyle factors (smoking, alcohol), relationship status and sexual activity, climacteric symptoms (including vasomotor symptoms, vaginal dryness, dyspareunia, low libido, anorgasmia, insomnia), surgical history (hysterectomy, oophorectomy), and self-reported depression. Use of menopause-related treatment was recorded, although treatment type (hormonal vs. non-hormonal) and route of administration were not consistently available.

HRQoL was assessed using the Cervantes SF-16 scale, which is a validated, menopause-specific patient-reported outcome measure developed to quantify the impact of menopausal symptoms on women’s HRQoL [13]. As a short-form version, it retains the conceptual structure of the original Cervantes scale while reducing respondent burden, which is particularly important in app-based data collection [14]. The instrument captures multidimensional aspects of the menopausal experience through four domain scores (Menopause–Health, Psychological, Sexuality, and Partner) and a global score, enabling both domain-specific and overall interpretation [15]. Use of a validated scale strengthens measurement reliability and comparability across studies, supporting the interpretability of observed group differences and minimizing misclassification of HRQoL outcomes. Items are rated on a 6-point Likert scale (0–5), with higher scores indicating worse HRQoL [Appendix A].

### 2.3. Statistics and Sample Size Estimation

Data were exported to a unified dataset (Microsoft Excel) and analyzed using MATLAB® v2024b (iOS). Continuous variables are presented as mean ± SD; normality was assessed with the Kolmogorov–Smirnov test. Between-group comparisons used Student’s *t* test or Mann–Whitney U test, as appropriate. Categorical variables are reported as *n* (%) and compared using χ^2^ or Fisher’s exact test. For comparisons across multiple gynecologic cancer subtypes, one-way ANOVA or Kruskal–Wallis tests were applied. Statistical significance was set at *p* < 0.05.

An a priori sample size calculation for independent means assumed SD = 20, a minimum detectable difference of 5 points, α = 0.05, a 3:1 allocation ratio (no cancer: cancer), and ≥80% power, with 10% inflation for invalid surveys (EPIDAT® 4.1).

## 3. Results

### 3.1. Study Population and Baseline Characteristics

**Figure 1:**
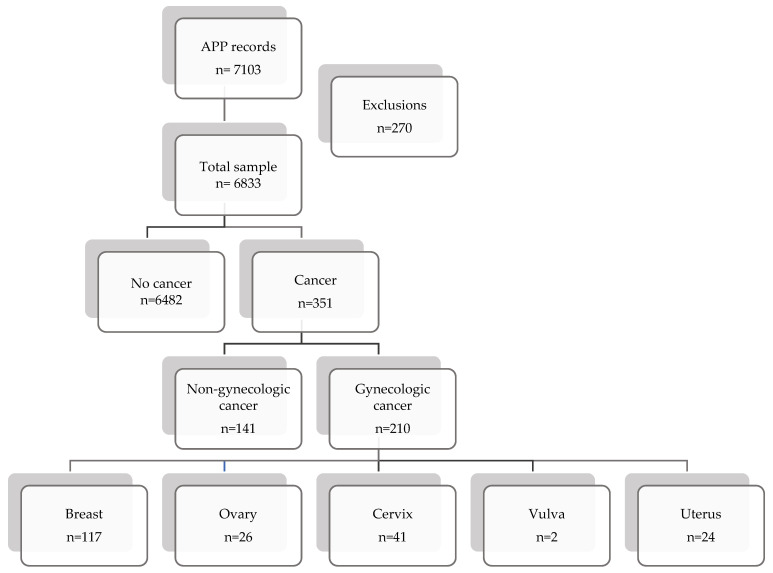
Flow diagram of the study sample. Records in the *Mi Menopausia* app (June 2021–June 2024): 7103; exclusions: 270 (incorrect/incomplete data, unclear responses, or duplicate entries). Analyzed sample: 6833 women. Group 1 (no cancer): *n* = 6482. Group 2 (cancer): *n* = 351 → non-gynecologic *n* = 141; gynecologic *n* = 210 (breast *n* = 117, ovary *n* = 26, cervix *n* = 41, vulva *n* = 2, uterus *n* = 24). Note: *n* = number of participants.

A total of 7103 app records were screened; 270 were excluded due to incorrect, incomplete, unclear, or duplicate entries, resulting in 6833 eligible peri-/postmenopausal women (Figure 1). Participants were classified as no cancer (*n* = 6482) or cancer (*n* = 351), including gynecologic (*n* = 210) and non-gynecologic cancers (*n* = 141).

Sociodemographic characteristics and lifestyle variables of the overall study population (*n* = 6833) are presented in Appendix B.

Baseline characteristics are summarized in Table 1. Comparing women with cancer with women without cancer, those with cancer were more frequently postmenopausal (71% vs. 51%, *p* < 0.01) and reported higher rates of hysterectomy (26% vs. 5%, *p* < 0.01) and oophorectomy (26% vs. 3%, *p* < 0.01), whereas age and BMI were similar between groups (Table 1). Climacteric symptoms were highly prevalent in both groups (96%).

### 3.2. Climacteric Symptom Profile

Climacteric symptoms were highly prevalent in both groups (Table 1). Symptom-level comparisons are shown in Table 2. Compared with women without cancer, those with cancer more often reported hot flushes (64% vs. 58%, *p* = 0.040), tremors (9% vs. 6%, *p* = 0.038), sweating episodes (37% vs. 29%, *p* = 0.001), dyspareunia (37% vs. 29%, *p* = 0.001), and anorgasmia (19% vs. 13%, *p* = 0.001), whereas irritability and low mood/easy crying were more frequent in the no-cancer group (*p* ≤ 0.016).

### 3.3. Menopause-Specific HRQoL (Cervantes SF-16): Cancer Versus No Cancer

Cervantes SF-16 domain and global scores are presented in Table 3. Women with cancer showed a significantly worse Sexuality domain score (mean ± SD was 48.3 ± 24.6 in women without cancer and 51.2 ± 23.8 in women with cancer; *p *= 0.013), while no statistically significant differences were observed in Menopause–Health, Psychological, Partner, or the global score.

### 3.4. Menopause-Specific HRQoL (Cervantes SF-16) by Cancer Category

Among women with cancer, HRQoL comparisons between gynecologic and non-gynecologic cancers are shown in Table 4. Non-gynecologic cancer was associated with worse Sexuality domain scores (55.7 vs. 48.2; *p* = 0.005), whereas other domains and global score did not differ significantly between cancer categories.

### 3.5. Exploratory Analysis by Gynecologic Cancer Subtype

Cervantes SF-16 domain scores by gynecologic subtype are summarized in Table 5. Significant between-subtype differences were observed in the Menopause–Health domain (the uterine cancer group showed worse HRQoL than the breast cancer group (52.4 ± 19.9 vs. 40.8 ± 19.0; *p* = 0.012) and the ovarian cancer group (52.4 ± 19.9 vs. 35.8 ± 19.2; *p* = 0.002) and in the Partner domain, with worse scores in the breast cancer group than in the ovarian cancer (21.4 ± 25.2 vs. 11.5 ± 17.6; *p* = 0.037). No significant differences were detected for Psychological, Sexuality, or global scores across subtypes (Table 5), but a non-significant trend was observed for the Global score when comparing breast versus ovarian cancer (*p* = 0.066).

### 3.6. HRQoL According to Menopause Treatment Use

Associations between menopause treatment use and HRQoL are shown in Table 6 and Table 7. In women without cancer, those reporting menopause-related treatment (any type of treatment; without specifying whether hormonal or non-hormonal, or route of administration) had higher Cervantes Scale scores, indicating worse HRQoL. Differences were statistically significant in the Menopause–Health domain (42.3 ± 19.4 vs. 40.3 ± 20.2; *p* = 0.002) and in the Psychological domain (44.3 ± 27.2 vs. 41.9 ± 28.1; *p* = 0.012). In the cancer group, HRQoL did not differ by treatment status (Table 6).

In stratified analyses, Sexuality scores differed by treatment status in opposite directions for non-gynecologic and gynecologic cancers: in women with non-gynecologic cancer, those reporting menopause-related treatment (any type of treatment; without specification of hormonal vs. non-hormonal therapy or route of administration), showed higher Sexuality domain scores, indicating worse sexual HRQoL (67.7 ± 22.4 vs. 53.4 ± 22.5; *p* = 0.007). In women with gynecologic cancer, the difference was also Sexuality, but in this case, scores were worse among women not receiving menopause-related treatment (49.8 ± 23.9 vs. 41.3 ± 23.8; *p* = 0.046). No significant differences in the other domains (Table 7).

### 3.7. HRQoL According to Depression Diagnosis

Depression was consistently associated with worse HRQoL (Table 8 and Table 9). In women without cancer, depression was associated with significantly worse scores across all domains and the global score (Table 8). In women with cancer, depression was also associated with worse HRQoL across domains except the Partner domain (Table 8). When stratified by cancer category, depression was associated with worse Menopause–Health, Psychological, and global scores in both non-gynecologic and gynecologic cancer groups (Table 9).

## 4. Discussion

This study used real-world data from a large app-based cohort to compare menopause-specific HRQoL, assessed with the Cervantes SF-16 scale, between peri- and postmenopausal women with and without a history of cancer. The main findings were: (1) the most notable difference between women with and without cancer occurred in the Sexuality domain, whereas global HRQoL scores were comparable; (2) within the cancer group, Sexuality scores were worse in non-gynecologic than in gynecologic cancers; (3) depression emerged as a strong and consistent correlate of poorer HRQoL across domains in both cancer and non-cancer groups; and (4) menopause-treatment use was associated with worse Menopause–Health and Psychological HRQoL among women without cancer.

The impairment observed in the Sexuality domain among cancer survivors suggests that a history of cancer may disproportionately compromise sexual wellbeing during the menopausal transition without necessarily resulting in a parallel decline across other HRQoL domains. Several mechanisms may explain the sexual HRQoL impairment among cancer survivors [7,16,17]: treatment-induced ovarian insufficiency and abrupt hypoestrogenism, dyspareunia, fatigue, and changes in self-image and relationship dynamics [5,16,17]. Sexual dysfunction has been highlighted as a clinically relevant issue in gynecological cancer care, supporting the need for systematic assessment and management [10,17,19]. By contrast, the absence of significant differences in global scores may reflect adaptation over time, heterogeneity in cancer types and treatments, and the possibility that non-sexual domains are influenced more strongly by shared determinants such as ageing, comorbidities, and menopausal status, thereby attenuating between-group differences in overall HRQoL [20].

The observation that Sexuality scores were worse in non-gynecologic than gynecologic cancers should be interpreted cautiously but is clinically meaningful. This counterintuitive pattern may reflect differences in age distribution, time since diagnosis, treatment exposures (e.g., systemic therapies, endocrine treatments), and survivorship trajectories that were not fully captured in the dataset [20,21]. Another plausible explanation is differential access to specialized care: women treated for gynecologic cancers are more likely to be followed in specialized women’s health settings where genitourinary symptoms and sexual concerns may be more routinely recognized and addressed [22]. Conversely, sexual health may receive less systematic attention in follow-up pathways for some non-gynecologic cancers, and these patients may more often receive intensive systemic treatments with broader physical and psychological sequelae, factors that could contribute to unmet needs and poorer sexual HRQoL [21,22,23].

Depression emerged as one of the most influential correlates of impaired HRQoL across the cohort. In both women with and without cancer, depression was associated with markedly worse scores across Cervantes SF-16 domains, consistent with evidence that mental health symptoms can be among the strongest drivers of perceived wellbeing and may overshadow differences attributable to cancer history alone [24,25]. When cancer survivors were stratified by tumor category, depression remained consistently associated with worse Menopause–Health, Psychological, and global HRQoL in both gynecologic and non-gynecologic cancers, whereas Sexuality and Partner domains did not show statistically significant differences in these subgroup analyses [24]. This may reflect limited statistical power, greater heterogeneity within subgroups, or stronger competing influences on sexual and partner-related outcomes (e.g., treatment sequelae, relationship status, sexual activity, and partner factors) [23,26,27,28]. Taken together, these findings highlight depression as both a critical confounder in observational analyses and a key clinical target within menopause and survivorship care pathways [29,30].

Finally, menopause-related treatment use was associated with worse Menopause–Health and Psychological HRQoL among women without cancer, an association that likely reflects confounding by indication: women who seek or receive treatment—whether hormonal or non-hormonal—typically do so because they have more severe symptoms [2,31,32,33]. In women with cancer, HRQoL did not differ by treatment status, possibly due to heterogeneous therapies, mixed indications, and stronger underlying determinants such as treatment-induced menopause and depression [4,10,34,35]. Subgroup analyses showed divergent associations in the Sexuality domain: among women with non-gynecologic cancer, those reporting menopause-related treatment had worse sexual HRQoL, whereas in gynecologic cancer survivors, worse Sexuality scores were observed among women not receiving treatment [36,37]. This apparently paradoxical pattern may reflect confounding by indication (treatment being more common in women with greater baseline symptom severity), differences in follow-up pathways, access to specialized gynecologic care, heterogeneity in treatment exposures and timing since cancer therapy [38,39].

**Clinical implications:** Our findings indicate that during the menopausal transition, a history of cancer is associated primarily with a sexual HRQoL burden, while depression contributes substantially and consistently to worse HRQoL across domains. These results suggest that integrating sexual medicine and mental health strategies into cancer survivorship and menopause services may yield meaningful improvements in patient-centered outcomes.

**Limitations:** Several limitations should be considered when interpreting these findings. First, the cross-sectional design prevents any causal inference and limits the interpretation of treatment-related associations. Second, participation was based on self-selection into an app-based cohort, introducing the possibility of selection bias, since users of a menopause-focused mobile application may differ systematically from the general target population in symptom burden, health awareness, digital literacy, or care-seeking behavior. Third, cancer history, treatments, depression diagnosis, and symptoms were self-reported, which may have resulted in misclassification, reporting bias, and social desirability bias. Non-response bias is also possible, as women who did not complete the questionnaires or provided incomplete data may have differed from those retained in the final sample. Fourth, the dataset lacked important clinical information, such as cancer stage, time since diagnosis, comorbidities, and detailed treatment characteristics. Finally, several subgroup analyses were based on small numbers, particularly for less frequent cancer types such as vulvar cancer, and should therefore be regarded as exploratory. Despite these limitations, the large sample size, real-world data capture, and use of a validated menopause-specific HRQoL measure provide important strengths and enhance the relevance of the findings.

## 5. Conclusions

In this large real-world cohort of peri- and postmenopausal women, cancer history was associated with worse sexual HRQoL, while global scores were comparable to those of women without cancer. Menopause-related treatment use was linked to poorer Menopause–Health and Psychological HRQoL only in women without cancer, likely reflecting confounding by indication whereby treatment use marks greater baseline symptom severity.

Depression showed a strong and consistent association with substantially worse menopause-related HRQoL, including women with and without a history of cancer, and across gynecologic and non-gynecologic cancer subgroups. Overall, these findings emphasize sexual wellbeing and mental health as key targets for integrated, patient-centered menopausal care in all menopausal women, with special relevance for cancer survivors [37,38,39].

## Figures and Tables

**Table 1:** Baseline characteristics of the sample with cancer versus women without cancer (mean ± SD or *n*, %).

	CANCER (*n* = 351)			NO CANCER (*n* = 6482)			*p*-Value
	*n*	%	Mean ± SD	*n*	%	Mean ± SD	
Age (years)	–	–	52.2 ± 7.9	–	–	51.7 ± 5.9	0.357
BMI (kg/m^2^)	–	–	24.9 ± 4.9	–	–	25.0 ± 5.3	0.880
Perimenopause	102	29	–	3173	49	–	<0.01
Postmenopause	249	71	–	3309	51	–	<0.01
Absence of climacteric symptoms	13	4	–	229	4	–	0.874
Presence of climacteric symptoms	338	96	–	6253	96	–	0.980
Hysterectomy	81	23	–	296	5	–	<0.01
Oophorectomy	81	23	–	148	3	–	<0.01
Depression	96	28	–	1429	24	–	0.130
Menopause treatment	61	17	–	1040	16	–	0.523
Antidepressants	7	2	–	59	1	–	0.044
Osteoporosis treatment	42	12	–	406	6	–	<0.01
No menopause treatment	290	83	–	5442	84	–	0.511

Note: *n* = number of participants; SD = standard deviation; BMI = body mass index. Subcategories of menopause treatment are not mutually exclusive and are shown as *n* (%) of the total group.

**Table 2:** Prevalence of climacteric symptoms in women with cancer versus women without cancer (*n*, %).

Symptom	CANCER (*n* = 351)	NO CANCER (*n* = 6482)	*p*-Value
Hot flushes	225 (64%)	3785 (58%)	0.040
Tremors	30 (9%)	378 (6%)	0.038
Sweating episodes	130 (37%)	1875 (29%)	0.001
Vaginal dryness	204 (58%)	3516 (54%)	0.174
Dyspareunia	130 (37%)	1875 (29%)	0.001
Low libido	198 (56%)	3688 (57%)	0.809
Anorgasmia	68 (19%)	866 (13%)	0.001
Insomnia	194 (55%)	3746 (58%)	0.320
Emotional irritability	139 (39%)	3038 (47%)	0.006
Low mood/easy crying	137 (39%)	2947 (45%)	0.016
Feeling of ageing	175 (50%)	3293 (51%)	0.689
Joint pain	222 (63%)	3889 (60%)	0.253

Note: *n* = number of participants.

**Table 3:** Menopause-specific HRQoL (Cervantes SF-16) domain and global scores in women with cancer versus women without cancer (Mean ± SD).

Cervantes SF-16 Domain	CANCER (*n* = 351)	NO CANCER (*n* = 6482)	*p*-Value
Menopause–Health domain	41.6 ± 20.9	40.6 ± 20.1	0.552
Psychological domain	41.2 ± 27.4	42.3 ± 27.9	0.524
Sexuality domain	51.2 ± 23.8	48.3 ± 24.6	0.013
Partner domain	19.9 ± 24.6	21.4 ± 23.9	0.090
Global score	30.6 ± 21.7	32.3 ± 20.7	0.130

Note: SD = standard deviation.

**Table 4:** Menopause-specific HRQoL (Cervantes SF-16) domain and global scores in gynecologic versus non-gynecologic cancer survivors (Mean ± SD).

Cervantes SF-16 Domain	GYNECOLOGIC CANCER (*n* = 210)	NON-GYNECOLOGIC CANCER (*n* = 141)	*p*-Value
Menopause–Health domain	42.6 ± 20.7	39.9 ± 21.1	0.227
Psychological domain	39.7 ± 27.3	43.5 ± 27.5	0.180
Sexuality domain	48.2 ± 24.1	55.7 ± 22.9	0.005
Partner domain	19.7 ± 24.9	20.1 ± 24.2	0.725
Global score	30.2 ± 21.7	31.3 ± 21.7	0.479

Note: SD = standard deviation.

**Table 5:** Cervantes SF-16 domain and global scores by gynecologic cancer subtype (Mean ± SD). Pairwise *p*-values for comparisons across gynecologic cancer subtypes.

Gynecologic Cancer Subtype	MENOPAUSE–HEALTH	PSYCHOLOGICAL	SEXUALITY	PARTNER	GLOBAL SCORE
Breast (*n* = 117)	40.8 ± 19.0	35.9 ± 25.1	50.8 ± 24.5	21.4 ± 25.2	31.1 ± 20.3
Ovary (*n* = 26)	35.8 ± 19.2	40.3 ± 24.8	45.0 ± 23.7	11.5 ± 17.6	23.1 ± 18.0
Cervix (*n* = 41)	45.2 ± 23.7	42.3 ± 29.1	43.7 ± 21.3	19.5 ± 23.5	32.2 ± 23.4
Vulva (*n* = 2)	39.1 ± 18.5	56.7 ± 14.1	50.0 ± 28.3	0.0 ± 0.0	20.4 ± 28.8
Uterus (*n* = 24)	52.4 ± 19.9	48.9 ± 33.3	46.3 ± 27.2	19.2 ± 29.2	28.7 ± 25.7
**Pairwise ** ***p*** **-values**					
**Subtype** **Comparisons**	**MENOPAUSE-HEALTH**	**PSYCHOLOGICAL**	**SEXUALITY**	**PARTNER**	**GLOBAL SCORE**
Breast vs. Ovary	0.172	0.379	0.484	0.037	0.066
Breast vs. Cervix	0.282	0.250	0.126	0.698	0.905
Breast vs. Vulva	0.983	0.209	0.941	0.210	0.555
Breast vs. Uterus	0.012	0.102	0.392	0.306	0.550
Ovary vs. Cervix	0.099	0.718	0.597	0.115	0.119
Ovary vs. Vulva	0.789	0.262	0.749	0.280	0.857
Ovary vs. Uterus	0.002	0.513	0.875	0.448	0.504
Cervix vs. Vulva	0.665	0.582	0.726	0.140	0.543
Cervix vs. Uterus	0.242	0.470	0.831	0.502	0.561
Vulva vs. Uterus	0.386	0.735	0.808	0.320	0.696

Note: SD = standard deviation.

**Table 6:** Menopause-specific HRQoL (Cervantes SF-16) domain and global scores by menopause treatment use in women with and without cancer (Mean ± SD).

Group	Treatment Status	MENOPAUSE–HEALTH	PSYCHOLOGICAL	SEXUALITY	PARTNER	GLOBAL SCORE
NO CANCER (*n* = 6482)	No menopause treatment (*n* = 5442)	40.3 ± 20.2	41.9 ± 28.1	48.3 ± 24.7	21.5 ± 24.2	32.3 ± 20.7
	Menopause treatment (*n* = 1040)	42.3 ± 19.4	44.3 ± 27.2	48.9 ± 24.4	20.5 ± 23.0	32.8 ± 20.4
*p*-value		0.002	0.012	0.446	0.176	0.457
CANCER (*n* = 351)	No menopause treatment (*n* = 290)	41.3 ± 20.7	40.3 ± 27.2	51.3 ± 23.4	20.2 ± 24.7	30.5 ± 21.7
	Menopause treatment (*n* = 61)	42.8 ± 22.0	45.8 ± 28.1	50.8 ± 26.4	18.2 ± 24.4	31.4 ± 21.9
*p*-value		0.607	0.153	0.893	0.563	0.758

Note: Data are presented as mean ± SD. Higher scores indicate worse health-related quality of life (HRQoL). “Menopause treatment” refers to any menopause-related therapy reported by participants; treatment type (hormonal vs. non-hormonal) and route of administration were not specified in this analysis.

**Table 7:** Menopause-specific HRQoL (Cervantes SF-16) domain and global scores by menopause treatment use in women with gynecologic versus non-gynecologic cancer (Mean ± SD).

Group	Treatment Status	MENOPAUSE–HEALTH	PSYCHOLOGICAL	SEXUALITY	PARTNER	GLOBAL SCORE
NON- GYNECOLOGIC CANCER (*n* = 141)	No menopause treatment (*n* = 119)	40.4 ± 21.2	43.2 ± 27.3	53.4 ± 22.5	19.3 ± 24.1	30.1 ± 21.7
	Menopause treatment (*n* = 22)	37.8 ± 21.2	45.8 ± 29.2	67.7 ± 22.4	24.1 ± 25.0	37.8 ± 21.3
*p*-value		0.597	0.683	0.007	0.398	0.130
GYNECOLOGIC CANCER (*n* = 210)	No menopause treatment (*n* = 171)	41.9 ± 20.4	38.3 ± 27.1	49.8 ± 23.9	20.8 ± 25.2	30.7 ± 21.8
	Menopause treatment (*n* = 39)	45.7 ± 22.2	45.8 ± 27.9	41.3 ± 23.8	14.9 ± 23.7	27.8 ± 21.7
*p*-value		0.313	0.120	0.046	0.180	0.458

Note: Data are presented as mean ± SD. Higher scores indicate worse health-related quality of life (HRQoL). “Menopause treatment” refers to any menopause-related therapy reported by participants; treatment type (hormonal vs. non-hormonal) and route of administration were not specified in this analysis.

**Table 8:** Menopause-related HRQoL (Cervantes SF-16) by depression status in women with and without cancer.

Group	Depression Status	MENOPAUSE–HEALTH	PSYCHOLOGICAL	SEXUALITY	PARTNER	GLOBAL SCORE
NO CANCER (*n* = 6482)	No depression (*n* = 5053)	38.3 ± 19.5	37.8 ± 26.5	47.3 ± 24.2	20.9 ± 23.2	31.3 ± 19.3
	Depression (*n* = 1429)	47.7 ± 20.0	56.8 ± 27.4	52.5 ± 25.2	22.8 ± 25.8	35.9 ± 23.8
*p*-value		<0.001	<0.001	<0.001	0.009	<0.001
CANCER (*n* = 351)	No depression (*n* = 255)	38.5 ± 19.5	35.6 ± 25.1	49.7 ± 23.6	18.8 ± 22.4	28.5 ± 19.7
	Depression (*n* = 96)	49.4 ± 22.5	56.6 ± 27.9	55.6 ± 23.9	23.3 ± 30.1	36.6 ± 25.8
*p*-value		<0.001	<0.001	0.037	0.131	0.002

Note: Data are presented as mean ± SD. Higher scores indicate worse HRQoL.

**Table 9:** Menopause-related HRQoL (Cervantes SF-16) by depression status in women with non-gynecologic and gynecologic cancer.

Group	Depression Status	MENOPAUSE–HEALTH	PSYCHOLOGICAL	SEXUALITY	PARTNER	GLOBAL SCORE
NON- GYNECOLOGIC CANCER (*n* = 141)	No depression (*n* = 101)	36.1 ± 19.2	36.2 ± 24.9	54.4 ± 21.3	19.9 ± 22.0	29.0 ± 19.7
	Depression (*n* = 40)	49.8 ± 22.6	62.2 ± 25.7	58.7 ± 25.0	22.2 ± 29.5	37.6 ± 25.7
*p*-value		<0.001	<0.001	0.308	0.609	0.036
GYNECOLOGIC CANCER (*n* = 210)	No depression (*n* = 149)	40.1 ± 19.7	35.2 ± 25.2	46.6 ± 24.5	18.1 ± 22.6	28.1 ± 19.7
	Depression (*n* = 61)	49.1 ± 22.7	52.6 ± 28.9	53.4 ± 23.1	24.1 ± 30.8	35.9 ± 26.0
*p*-value		0.006	<0.001	0.073	0.130	0.021

Note: Data are presented as mean ± SD. Higher scores indicate worse HRQoL.

## Data Availability

The data supporting the findings of this study are not publicly available due to privacy and data protection requirements related to the app-based dataset, but may be made available from the corresponding author upon reasonable request and subject to appropriate approvals and data-sharing agreements.

## Abbreviations

The following abbreviations are used in this manuscript:

AEEM	Spanish Association for the Study of Menopause (*Asociación Española para el Estudio de la Menopausia*);
BMI	Body Mass Index
HRQoL	health-related quality of life;
SD	standard deviation

## Appendix A

**Table A1:** Cervantes SF-16 scale questionnaire (English version; Likert 0–5).

MENOPAUSE-HEALTH DOMAIN	1. Suddenly, I notice that I start sweating even though I have not made any effort.	Never	0	1	2	3	4	5	Always
	2. I notice hot flushes.	Never	0	1	2	3	4	5	Always
	3. During the day, I notice that my headache progressively worsens.	Never	0	1	2	3	4	5	Always
	4. Even though I sleep, I do not feel rested.	Never	0	1	2	3	4	5	Always
	5. I notice that my heart beats very fast and uncontrollably.	Never	0	1	2	3	4	5	Always
	6. I feel tingling in my hands and feet.	Never	0	1	2	3	4	5	Always
	7. I am afraid to make physical efforts because I may leak urine.	Never	0	1	2	3	4	5	Always
	8. My health causes problems with daily activities (housework, going to work, etc.)	Never	0	1	2	3	4	5	Always
	9. I have noticed that my skin is drier.	Never	0	1	2	3	4	5	Always
PSYCHOLOGICAL DOMAIN	10. I cannot take it anymore because of how nervous I feel.	Never	0	1	2	3	4	5	Always
	11. Everything bores me, even things that used to be fun.	Never	0	1	2	3	4	5	Always
	12. From the moment I get up, I feel tired.	Never	0	1	2	3	4	5	Always
SEXUALITY DOMAIN	13. In my life, sex is…	Not important	0	1	2	3	4	5	Very important
	14. I am satisfied with my current sexual activity (even if I have none)	Never	0	1	2	3	4	5	Always
	If you do not currently have a partner, do not answer the following questions.								
PARTNER DOMAIN	15. I consider myself happy in my relationship.	Not at all	0	1	2	3	4	5	Very much
	16. My role as a wife or partner is…	Not important	0	1	2	3	4	5	Very important

## Appendix B

**Table A2:** Sociodemographic characteristics and lifestyle variables of the study population (*n* = 6833).

VARIABLE	*n* = 6833	%
**Alcohol consumption**		
• None	887	13%
• Occasional	3237	47%
• <3UI/week	1610	24%
• ≥3UI/week	1099	16%
**Smoking status**		
• None	4638	68%
• Occasional	327	5%
• Daily	949	14%
• Former smoker	919	13%
**Sexual activity**		
• None	647	9%
• ≥1 per month	3774	56%
• Occasional	1706	25%
• Masturbation only	611	9%
• Caressing only	95	1%
**Relationship status**		
• Single	905	13%
• Non-stable partner	254	4%
• Stable partner	5674	83%
**Employment status**		
• Homemaker	628	9%
• Employed outside home	5525	81%
• Unemployed	411	6%
• Retired/Disabled	269	4%
**Educational level**		
• No formal education	73	1%
• Primary education	777	11%
• Secondary education	1912	28%
• University education	4071	60%
**Continent of origin**		
• America	615	9%
• Europe	6172	90%
• Africa	14	0.3%
• Asia	20	0.3%
• Oceania	12	0.3%
**Town/city size (inhabitants)**		
• <30,000	1802	26%
• 30,000–100,000	1779	26%
• >100,000	3252	48%
